# The interaction between subclinical psychotic experiences, insomnia and objective measures of sleep

**DOI:** 10.1016/j.schres.2017.06.058

**Published:** 2018-03

**Authors:** Jan Cosgrave, Ross Haines, Dalena van Heugten-van der Kloet, Ross Purple, Kate Porcheret, Russell Foster, Katharina Wulff

**Affiliations:** a Sleep and Circadian Neuroscience Institute, Nuffield Department of Clinical Neurosciences, University of Oxford, Oxford, United Kingdom; b Department of Statistics, University of Oxford, United Kingdom; c Oxford Brookes University, Faculty of Health and Life Sciences, Department of Psychology, Social Work and Public Health, Oxford, United Kingdom

**Keywords:** Insomnia, Psychotic-like experiences, Actigraphy, Circadian timing, Psychosis

## Abstract

Investigations into schizophrenia have revealed a high incidence of comorbidity with disturbed sleep and circadian timing. Acknowledging this comorbidity on a dimensional level, we tested prospectively whether subclinical psychotic symptoms are more prevalent in individuals with insomnia. An insomnia group (*n* = 21) and controls (*n* = 22) were recruited on their subjective sleep quality, recorded actigraphically for 3 weeks and assessed for psychotic-like experiences with The Prodromal Questionnaire-16. Using multivariate Poisson regression analyses, we found that objective and subjective sleep measures interact to predict the highest risk for psychotic experiences. Objective measures of sleep and statistical modelling are rarely used in either clinical trials or practice for schizophrenia, yet this study highlights their value in these areas.

## 1 Introduction

Evidence is now accruing that sleep and circadian rhythm disruption (SCRD) is a ubiquitous feature of psychosis. A study by Cohrs (2008) highlighted that between 30% and 80% of patients with a diagnosis of schizophrenia report sleep disturbances (SD). This frequently manifests as patients exhibiting a range of SCRD related phenotypes, ranging from extended time to get to sleep (sleep onset latency), difficulties with sleep continuity (with a prevalence of 50–70%; Benson, 2006, Tandon et al., 2008, Waters and Manoach, 2012) to extreme circadian misalignment (sleep phase advances/delays, bidian cycles and non-24 h periods) and highly irregular and fragmented sleep patterns (Wulff et al., 2012). These disturbances are also associated with important clinical outcomes, including relapse (Waters and Manoach, 2012), poorer coping (Ritsner et al., 2004), higher distress (Hofstetter et al., 2005), and increased frequency of depression (Palmese et al., 2011) and completed suicide (Pompili et al., 2009).

It has been debated that SCRD may represent a vulnerability factor, or be involved in the development of psychosis, on the understanding that sleep/circadian abnormalities and the aetiology of psychosis are rooted in failures of genetic and synaptic functions of neurotransmitter systems (e.g. GABA, glutamate, dopamine) and neural/humoral circuits (e.g. hypothalamic-pituitary-adrenal axis; see reviews; Pritchett et al., 2012, Wulff et al., 2010). This is evidenced by the omnipotence of poor sleep across all of the core phases of the disorder, including the prodrome (with an estimated prevalence of 70–100%; Yung and McGorry, 1997), as well as acute (Kupfer et al., 1970), chronic, and residual phases (Waters et al., 2011).

More recently, the notion of a direct link between sleep/circadian dysfunction and the development of psychosis has been further addressed by examining of the presence of SCRD prior to the occurrence of psychotic episodes (see review Lunsford-Avery and Mittal, 2013). While there was overwhelming evidence for a link, the majority of` data reviewed were subjective reports of sleep disturbance and only one published study had examined sleep in at-risk population using objective EEG methods (Keshavan et al., 2004). There are now two comprehensive systematic reviews examining the evidence to date in support of this relationship (Davies et al., 2016, Reeve et al., 2015). Davies et al. (2016) concluded that evidence to support the reliability of the observed association is limited due to considerable heterogeneity in samples and methods (non-affective, affective psychosis, ‘other psychosis’, prospective, retrospective, polysomnography, actigraphy, self-report, interview, etc.) and that the prevalence and nature of sleep disturbances cannot be defined as yet in ultra high-risk population. Nevertheless, the review highlights that poor sleep is related to greater severity in positive symptoms (Lunsford-Avery et al., 2015), higher distress (Andriopoulos et al., 2011) and, although unspecific, one out of six components in a prediction model for psychosis (Ruhrmann et al., 2010). Furthermore, there is also evidence that there may be a shared genetic and environmental lineage underlying psychotic experiences and SCRD (Taylor et al., 2015).

The differences observed in the prodromal phase equate to what is observed when the individual has transitioned to psychosis: extended sleep onset latency, difficulties with sleep continuity and lower circadian rest-activity amplitude (Castro et al., 2015, Zanini et al., 2013). It has also been noted that circadian phenotypic variation over 5 days of activity monitoring (lower daily activity, fragmented sleep patterning/misalignment with the light-dark cycle) predicted more severe psychotic symptoms and greater psychosocial impairment at a one-year follow-up in an adolescent cohort deemed clinically high-risk for psychosis (Lunsford-Avery et al., 2017). The authors concluded that circadian rhythm regulation might be a potential target for identification and interventions to stabilise social, eating and sleep-wake rhythms in early intervention services.

One of our goals continues to be the identification and characterisation of physiological sleep factors that impact mental function on an individual level. Given objective sleep differences have already been noted in individuals at risk of psychosis when compared to healthy controls (ex: Castro et al., 2015), it may then be more pertinent to note whether the signal is bi-directional: i.e. does a cohort with a complaint of poor sleep (insomnia) endorse a greater number of PLEs, and if so, what parameters of their sleep dictate this? We therefore tested whether the relationship between schizophrenia and sleep is bi-directional and hypothesised that healthy young adults with self-reported insomnia endorse a greater number of psychotic-like experiences than those with self-reported good sleep. We applied statistical models to examine whether parameters of sleep predict this relationship, and if so, which parameters: subjective, objective, or both.

A student sample was chosen because research on PLEs in this population may hold greater clinical relevance and these experiences may incur greater distress when compared to younger children and adolescents (Kelleher et al., 2012, Zammit et al., 2013). Furthermore, understanding sleep's role in PLEs is particularly pertinent in university students as they are a cohort particularly susceptible to disrupted sleep scheduling and day-time activity patterns (Brown et al., 2002).

## 2 Method

This sample included 43 healthy young adults (18–30 years), recruited from the University of Oxford and Oxford Brookes University. The Insomnia group were required to have a Pittsburgh Sleep Quality Index (PSQI) of 8 or above and an Insomnia Severity Index (ISI) of 10 or above, whilst controls were required to have a PSQI of 3 or below and an ISI of 6 or below, thereby creating a degree of separation in the groups' subjective reporting of sleep quality.

The PSQI (Buysse et al., 1989) measures subjective sleep quality over the previous month and yields a score ranging from 0 to 21, with higher scores representing poorer quality. The standardised cut-off score for poor sleep quality is 5. The ISI (Bastien, 2001) measures both night-time and day-time elements of insomnia, and ranges from 0 to 28. Scores of 10 and above are considered optimal for detecting insomnia in community samples (Morin et al., 2011). Both measures have shown good psychometric properties for use in both patients and healthy controls (Backhaus et al., 2002, Carpenter and Andrykowski, 1998, Morin et al., 2011).

Exclusion criteria for all participants included a diagnosis of a psychotic disorder (past or present); taking medication known to affect sleep; brain injury; epilepsy; shift work; hospitalisation in the previous six months; and travelling through two or more time zones in the previous fortnight. The study protocol was approved by the Medical Sciences Interdivisional Research Ethics Committee (MSD-IDREC-C1-2014-177) and all participants gave written informed consent.

As subjective sleep quality can reflect different sleep related experiences (ex: difficulty falling asleep, difficulty staying asleep) for different people and is correlated with non-sleep related phenomena (ex: mood; Krystal and Edinger, 2008), the sleep-wake cycle was objectively monitored for three weeks using wrist-worn actigraphs with an integrated light sensor (MotionWatch 8, CamNtech Ltd.). This was used in conjunction with a standardised diary of sleep timings and daily activities that was used to annotate the actigraphy data. Actigraphy data were sampled at one-minute epochs, and MotionWare software (version 1.1.15, CamNtech, Ltd.) was used to calculate sleep fragmentation (an index derived from the frequency and intensity of physical movement during the sleep period), sleep onset latency (SOL; the amount of time between bedtime and sleep onset), wake after sleep onset (WASO; the amount of time spent above a predefined activity threshold), total sleep time (TST; time between sleep onset and final wake time, excluding WASO), and variability in sleep onset and sleep duration (measured by their standard deviations).

Psychotic experiences were measured using the Prodromal Questionnaire 16 Item Version (PQ-16), which has acceptable psychometric properties in both healthy and high-risk populations (Ising et al., 2012). It contains 16 yes/no items, yielding a score out of 16. Scoring 5 or above warrants further screening for an at risk mental state (Ising et al., 2012). The Questionnaire assesses positive symptoms (visual and auditory hallucinations, delusional mood/perplexity, ideas of reference and persecutory thoughts), negative symptoms (excessive social anxiety) and avolition.

All measures were examined for their distributional properties. Actigraphic variables met the assumptions for parametric testing, as such Welch's two sample *t*-test (with continuity correction) was employed, and means were reported. All *p*-values reported were corrected for multiple testing using the Benjamini & Hochberg correction method (Benjamini and Hochberg, 1995). This correction controls for false discovery rate, as opposed to the more commonly employed Bonferroni method, which controls for the family-wise error rate.

## 3 Results

The sample comprised of 43 students: 21 in the insomnia group (mean age = 23.9 years, SD = 3.6, 13 women) and 22 controls (mean age = 22.8 years, SD = 3.2, 11 women). The mean PSQI of the insomnia group was 10.1 (SD = 2.2) compared to 2.4 (SD = 0.8) for the controls. The mean ISI was 14.4 (SD = 3.3) for the insomnia group compared to 1.3 (SD = 1.3) for the controls (Table 1). Independent-samples *t*-tests highlighted no significant differences between groups in any of the actigraphic measures taken after correcting for multiple comparisons with the exception of sleep period (time spent in bed excluding sleep onset latency; t(38.3) = − 3.02; *p* = 0.040) using the Benjamini Hochberg correction method.

**Table 1:** Descriptive statistics of the objective and subjective measures of sleep.

	Good Sleepers (*N* = 22)		Insomnia Group (*N* = 21)		Group Differences			
	Mean	(95% CI)	Mean	(95% CI)	t	df	p	p_adj_
Subjective Sleep								
PSQI	2.4	(1.99–2.74)	10.1	(9.23–11.22)	–	–	–	–
ISI	1.3	(0.75–1.89)	14.4	(13.13–16.16)	–	–	–	–
Objective Sleep (measured actigraphically over three weeks)^a^								
Sleep Onset (h:m)	00:58	(00:36–01:19)	00:59	(00:38–01:22)	− 0.14	42.0	0.892	0.892
Sleep Offset (h:m)	08:55	(08:32–09:18)	09:28	(09:02–09:53)	− 1.98	41.7	0.054	0.153
Sleep Period (hr)^b^	7.96	(7.68–8.24)	8.47	(8.26–8.67)	− 3.02	38.3	0.004	0.040
TST (hr)	6.69	(6.43–6.96)	6.95	(6.70–7.21)	− 1.48	42.0	0.147	0.184
SOL (min)	9.0	(6.6–11.4)	12.6	(9–16.8)	− 1.73	35.9	0.092	0.153
Fragmentation	27.58	(24.39–30.78)	31.33	(28.61–34.04)	− 1.86	40.9	0.071	0.153
WASO (hr)	1.27	(1.09–1.44)	1.51	(1.34–1.67)	− 2.10	42.0	0.042	0.153
SD Sleep Onset (hr)	1:05	(0:55–1:14)	1:16	(0:59–1:33)	− 1.22	32.1	0.233	0.259
SD Sleep Offset (hr)	01:18	(01:08–01:28)	01:33	(01:19–01:47)	− 1.79	38.2	0.081	0.153
Sleep Efficiency (%)	84.16	(82.11–86.21)	82.09	(80.04–84.14)	1.48	42.0	0.146	0.184

a Three individuals were excluded due to non-compliance (*n* = 1) and malfunctioning watches (*n* = 1) and suspected circadian rhythm disorder (*n* = 1).

b Sleep Period is the time spent in bed excluding SOL.

**Fig. 1:**
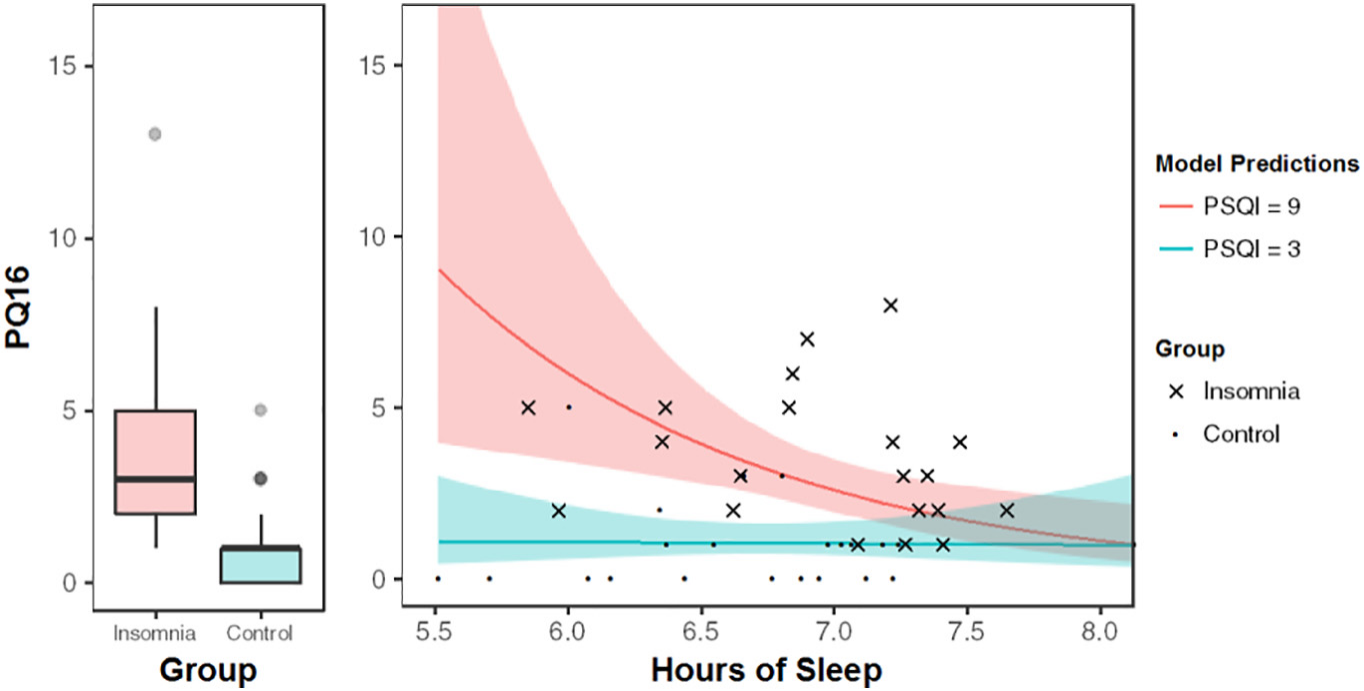
Left*:* Mean number of psychotic experiences endorsed on the PQ-16 by the insomnia and control groups. Error bars represent the standard error in each group. Right*:* The interaction between PSQI (subjectively perceived sleep quality) and hours of sleep (objective). Number of psychotic-like experiences endorsed on the PQ-16 (y-axis) against hours of sleep (x-axis, total sleep time as assessed by actigraphy). The predicted rates of psychotic experiences are shown for insomnia with perceived poor sleep (red) and controls with perceived good sleep (blue). Lower hours of sleep alone are not predictive for psychotic-like experiences, as indicated by the blue line, but are in combination with perceived poor sleep quality (red line). The shaded areas around each represent 95% confidence intervals.

A Wilcoxon Rank-Sum test indicated that the median PQ-16 score in the insomnia group (median = 3) was significantly higher than the control group (median = 1, 95% CI [1.00–4.00], W = 70.5, *p* < 0.001, Fig. 1).

Multivariate Poisson regression analyses were used to investigate which parameters of sleep best predicted the difference in psychotic experiences (PQ-16) between groups. Possible predictor variables for PQ-16 were PSQI, ISI, WASO, TST, fragmentation, SOL, and variability in both sleep onset and duration. Due to concerns with collinearity, PSQI and ISI were not included in models together.

Model selection was based upon Akaike's Information Criterion (AIC), which measures the relative quality of a collection of models (Bozdogan, 1987). Standard automated model selection procedures (forward selection and backwards elimination) were used to propose candidate models. Beginning with a simple model with no predictor variables, forward selection iteratively adds the predictors offering maximal AIC reduction until no further reduction is possible. Backward elimination instead iteratively removes predictor variables from a complex model, until no further AIC reduction is possible.

Our best model of fit included an interaction between PSQI (the subjective measure) and TST (objective measure) (see Table 2; β = − 0.13, SE = 0.05, z = − 2.58, *p* < 0.01). A graphical representation of the interaction can be seen in Fig. 1B. The solid blue line represents the predicted rate of psychotic experiences with a self-perception of good sleep (PSQI score of 3), whereas the solid red line shows the equivalent with a self-perception of poor sleep (PSQI score of 9). This highlights that the impact of TST and subjective sleep quality on psychotic experiences is different for the insomnia group as opposed to the control group.

**Table 2:** Summary of Model Output employing PQ16 as the Outcome Measure (*n* = 42)^a^.

Predictor Variable	β	SE	Z	p
Intercept	− 2.98	3.16	− 0.95	0.34
PSQI	1.07	0.34	3.13	< 0.01
TST	0.35	0.46	0.76	0.45
PSQI*TST	− 0.13	0.05	− 2.58	< 0.01

PSQI: Pittsburgh Sleep Quality Index; TST: Total Sleep Time (as measured by actigraphy).

a One participant was excluded due to incomplete questionnaire data.

**Fig. 2:**
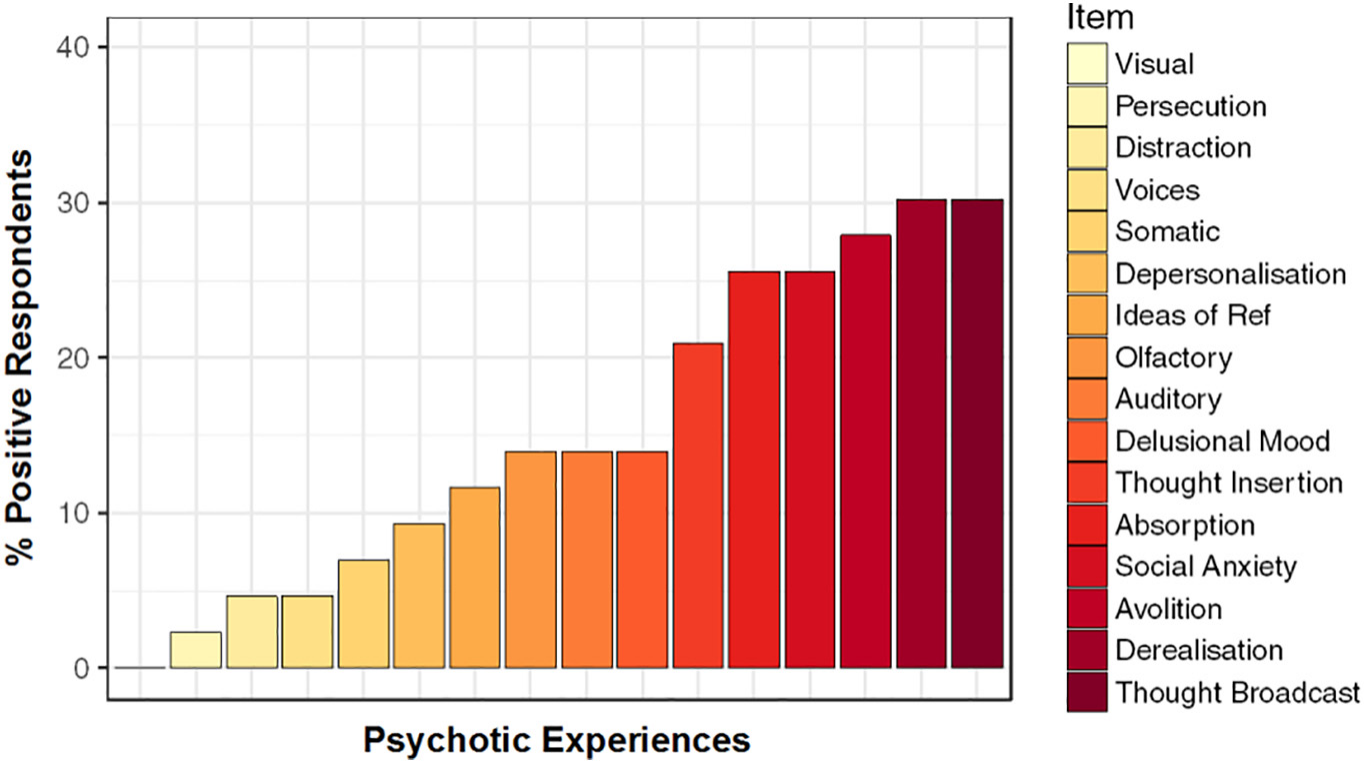
Distribution of items endorsed on the PQ16 (*n* = 42). Social anxiety and avolition are amongst the most highly endorsed items on the PQ16, however, thought broadcasting and derealisation are more frequently endorsed in this cohort.

Two items of the PQ16 refer to avolition (akin to depression) and social anxiety (Ising et al., 2012). As a means of verification to ensure these items were not overly influential on the model, we examined the breakdown of positive responses to PQ16 items across the cohort (Fig. 2). This distribution highlights that while avolition and social anxiety are amongst the highest scoring items in the questionnaire, they are still lower than derealisation and thought broadcasting, and are on parity with absorption - all of which are considered core symptoms of psychosis.

To further ensure that these two items did not overly influence the modelled relationship, the modelling process was repeated with the items excluded from the PQ16 score of each participant (Table 3). This highlighted an additional main effect of standard deviation of sleep onset (β = 0.58, SE = 0.21, z = 2.71, *p* < 0.01). This suggests that the variability in the onset of sleep is also important in the prediction of PEs, with greater variability predicting a greater PQ16 score.

**Table 3:** Poisson regression model with ‘PQ14’ as the outcome measure – specifically with avolition and social anxiety items removed from the PQ16 score (*n* = 42). The standard deviation of the sleep onset now features.

Predictor Variable	β	SE	Z	p
Intercept	− 2.43	3.68	− 0.66	0.51
PSQI	1.22	0.42	2.93	< 0.01
TST	0.15	0.54	0.28	0.78
St. Dev. Sleep Onset	0.58	0.21	2.71	< 0.01
PSQI*TST	− 0.15	0.06	− 2.48	0.01

## 4 Discussion

In a young healthy student sample, using both objective and subjective sleep measures of poor and good sleepers, we found an interaction between these measures in the prediction of psychotic-like experiences. If sleep quality is perceived as good, our model suggests a perception of good quality of sleep offers sufficient protection from psychotic-experiences, even with sleep below 7 h per night. If sleep quality is perceived as poor but objectively of substantial length (above 7.5 h), the risk of psychotic-like experiences is negligible: the same as with a perception of good sleep; however, with decreasing hours of sleep, this risk progressively starts to increase. Therefore, accounting for social anxiety and depressive symptoms, we find the combination of perceived poor sleep and an actual lack of sleep predicts the greatest risk in psychotic experiences.

Studies to date report associations between subjective measures of sleep and psychotic-like experiences in community samples (Taylor et al., 2015) and highlight that shared environmental influences and genetic lineage contribute to these associations. However, our study is the first to consider how objective, actigraphy-derived measures and data modelling can be implemented to increase our understanding of this relationship. Despite sounding intuitive, this concomitance of biological and psychological factors of sleep has not been reported in the context of psychotic symptomatology before. Furthermore, there is currently strong overreliance on subjective sleep measures, including the PSQI, in assessing risk in psychiatric populations (Buysse et al., 2006, Fairholme et al., 2015). Sleep quality questionnaires may not only capture sleep quality but also distress and anxiety in individuals with psychiatric comorbidity, due to retrospective recall bias as has recently been shown by Hartmann and colleagues (Hartmann et al., 2015). Given this psychological bias and the enormous variation in sleep-wake patterns in humans, our findings reinforce the necessity for both subjective and objective sleep measures in clinical trials and practice when investigating psychotic symptomatology. Our research also highlights that patients with an insomnia complaint married with objectively poorer sleep may indicate a more aggressive form of the disorder and may merit a separate targeted sleep treatment, or indeed indicate a heightened risk for individuals endorsing PLEs.

A number of caveats merit mention. First, self-report measures are open to bias due to participants having a lower threshold for complaints or a more liberal response style. This is particularly relevant for PLEs (Rossi et al., 2016). Second, while actigraphy is a commonly used longitudinal objective proxy measurement for the sleep-wake cycle, polysomnography is still considered the gold standard for determining an individual's total sleep time and overall sleep architecture (Krystal and Edinger, 2008). Third, with smaller samples, there is an increased risk of Type II errors (Batterham and Hopkins, 2006). Models for this study were selected on AIC values to negate an overreliance on *p*-values where possible to combat this. However, this nonetheless should be considered when interpreting the results.

These caveats notwithstanding, our results provide a starting point for further research. Future studies should aim to test the predictive power of this model by replicating the findings across larger community samples and different groups at risk of psychosis, thereby aiming to understand if the model can be generalized - or indeed, how it differs along the affective-psychosis continuum, unbiased by pre-defined clinical categories. Furthermore, it is unlikely that sleep and circadian rhythm disturbance will unilaterally impact or exacerbate all psychotic symptoms equally. What is more probable is that SCRD will have greater ties to certain psychotic symptoms and be less relevant for others. To further disentangle this complex relationship and to be able to answer more nuanced questions relating to the sleep-PLE relationship, future work should consider high-resolution sampling and longitudinal designs in both prodromal and insomnia populations to see if the signal is seen in both directions or how it differs.

## Role of the funding source

The lead author on this manuscript (JC) was funded by both the Medical Research Council and St. John's College, Oxford. The study was partly supported by the National Institute for Health Research (NIHR) Oxford Biomedical Research Centre based at Oxford University Hospitals NHS Trust, Oxford University (A90305 and A92181 to KW and RGF). The views expressed are those of the author(s) and not necessarily those of the NHS, the NIHR, or the Department of Health. This study was supported by the infrastructure of the Sleep and Circadian Neuroscience Institute (SCNi) (098461/Z/12/Z).

## Contributors

Jan Cosgrave collaboratively designed the protocol, collected and analysed the data and wrote the initial draft of the manuscript. Ross Haines collaboratively undertook and verified the statistical analyses and extensively edited the original draft of the manuscript. Dalena van Heugten-van der Kloet collaboratively designed the protocol and extensively edited the manuscript to its final version. Russel Foster, Ross Purple and Kate Porcheret collaboratively designed the protocol and extensively edited the manuscript to its final version. Katharina Wulff was integral to the development, design, supervision and writing of the manuscript. She extensively edited the manuscript and contributed copious amounts of knowledge and expertise to the manuscript and research as a whole. All authors contributed to and have approved the final manuscript.

## Conflict of interest

All authors declare that they have no conflicts of interest.
